# Pattern of Adolescent Suicides in Pakistan: A content analysis of Newspaper reports of two years

**DOI:** 10.12669/pjms.39.1.6851

**Published:** 2023

**Authors:** Nazish Imran, Sadiq Naveed, Bariah Rafiq, Sania Mumtaz Tahir, Maryam Ayub, Imran Ijaz Haider

**Affiliations:** 1 Nazish Imran, MBBS, FRCPsych (London), MRCPsych (London), MHPE, PhD. Professor, Child & Family Psychiatry, King Edward Medical University, Lahore Pakistan; 2 Sadiq Naveed, MD, Psychiatry Program Director, Eastern Connecticut Health Network, CT, United States; 3 Bariah Rafiq, MBBS. Postgraduate Psychiatry Resident, Academic Dept. of Psychiatry and Behavioral Sciences, King Edward Medical University, Lahore. Pakistan; 4 Sania Mumtaz Tahir, MBBS. Postgraduate Psychiatry Resident, Academic Dept. of Psychiatry and Behavioral Sciences, King Edward Medical University, Lahore. Pakistan; 5 Maryam Ayub, MBBS. Postgraduate Psychiatry Resident, Academic Dept. of Psychiatry and Behavioral Sciences, King Edward Medical University, Lahore. Pakistan; 6 Imran Ijaz Haider, FRCPsych, MRCPsych, DPM (UK). Professor of Psychiatry, Fatima Memorial Hospital, Lahore Pakistan

**Keywords:** Suicide, Children, Adolescents, Suicidal behaviors, Risk factors, Hanging

## Abstract

**Background and Objective:**

Suicide is a serious, yet preventable global mental health problem for people of all ages and countries. It is the third leading cause of death in 15-19-year-olds. There is paucity of systematic studies and official statistics on child & adolescent suicides in Pakistan. In the absence of other means, newspaper coverage of adolescent suicides may serve as a useful source to understand the phenomena. Our objective was to report pattern of suicide deaths in children and adolescents across Pakistan and to determine the predominant methods of suicide precipitating events and associated factors

**Methods:**

This study used content analysis to analyze newspaper reports of adolescent suicide in four leading newspapers of Pakistan from January 1^st^, 2019, through December 31^st^, 2020. Search yielded 289 child and adolescent (ages<18) suicide reports. Data about various sociodemographic characteristics, methods of suicide, possible motives, and associated features (e.g., any suicide notes) was extracted and analyzed.

**Results:**

Total 289 suicides in children and adolescents in Pakistan were reported in selected newspapers during two years among both genders (51.5 % boys and 48.5% girls) with high incidence in late adolescence (66%). The predominant method of suicide in this group was ingestion of poisonous substances (50%) followed by hanging (35%) and use of firearms (7%). The behavior usually takes place within or near the adolescent’s home environment. The act was often attributed to domestic conflicts including arguments with parents of the deceased and relationship problems.

**Conclusion:**

To develop effective suicide prevention strategies for a population, it must be studied within its own socio-cultural context. Study results emphasize adolescent suicide being a reality in Pakistan. There is urgent need for further culture specific research in this area in the country.

## INTRODUCTION

Suicide is a global public and mental health problem accounting for over 800,000 deaths worldwide.^1^ Suicidal behaviors are rare in childhood. However, there is an alarmingly higher vulnerability in adolescents with suicide being the third leading cause of death in 15-19 year-olds.^2^ Most (90%) adolescents, who died by suicide were from low- and middle-income countries (LMIC).^2^ A pooled suicide rate of 3.77 per 100,000 is reported in a recent meta-analytic review of worldwide suicide rates in adolescents.^3^ Thus, altogether adolescence may be considered as a key development period for effective suicide prevention and intervention.

Adolescence (10-19 years of age) is a time of significant physical, psychological, cognitive, and socio-emotional changes. Apart from exposure to recent pandemic related stressors, complex cultural, societal, family, environmental and economic factors in adolescence can have an adverse impact on their social and emotional well-being including increased risk of onset of mental illness and suicidal behaviors.^4^-^6^ Various risk factors for suicide in adolescence can be presence of psychiatric illness, substance abuse, previous suicide attempts, family history of suicide and suicidal and homicidal ideations.^7^

Over the last few decades, a number of studies involving children and adolescents from many countries suggested significant differences in the prevalence and epidemiological pattern of suicidal behaviors across age, gender, race, countries, and cultures.^2^ A global estimate for suicide mortality in early adolescents (10-14 years old) is 1.52 /100,000 for boys and 0.94/100,000 for girls but increase rapidly to 10/100,000 during late adolescence.^8^,^9^ Although these statistics appears staggering, they are just the tip of the iceberg as stigma, social taboos and legal issues around suicide reporting around the world may significantly underestimate the suicide problem in children & adolescents.

In Pakistan, almost 13% of the country’s 221 million population falls in the age group 10-14 years and 10.37% in 15-19 years of age.^10^ The real burden of mental health problems in children and adolescents in Pakistan is unknown due to lack of epidemiological research. There are no official national statistics regarding suicide in Pakistan, as it lacks vital statistics registration systems. Hence, data on suicidal behavior in Pakistani youth is also sparse.^11^ Demographic characteristics, family structure and presence of or history of psychiatric symptoms and suicide attempt in family or the individual himself are noted to contribute towards risk of suicide.^12^

Differences in pattern and characteristics of suicide among children and adolescents necessitates the analysis of suicidal deaths in younger age groups. Furthermore, awareness of trends, risk factors, and preferred methods of suicide in the country would also be helpful to extend our understanding of the problem and for planning prevention intervention for suicidal adolescents based on local evidence.

Absence of reliable national statistics in Pakistan makes media reports (despite various limitations) as a useful source of information for suicidal deaths. Thus, the present study set out to conduct a situational analysis of child & adolescent suicide in Pakistan through analysis of newspaper reports. Our objective was to report pattern of suicide deaths including age and gender in children and adolescents across Pakistan and to determine the predominant methods of suicide used by this age group, precipitating events and associated factors.

## METHODS

As part of a larger project, after Institutional Review Board approval (Ref 615/RC/KEMU), we undertook search for all news reporting on suicide deaths in four Pakistani newspapers three English Language (Daily Dawn, Daily Nation, and Daily Tribune) and one Urdu Language (Daily Jang) over the two-year period (i.e. January 1, 2019-December 31^st^, 2020). The four selected newspapers are among the topmost circulated newspapers in the country and comprehensively represent most of the influential publishing houses of country with credibility among readers. A pilot search of all four newspapers for a month revealed that among English newspapers, most but not all of suicides reported in The Nation (five cities’ editions) and Tribune (three cities editions) were also reported in Dawn (four cities editions). However, a significant proportion of suicides reported in the Urdu newspaper Jang (five local cities editions) were not reported in any of the English newspapers. Due to limited resources and time, and limited archives availability of online editions of other Urdu newspapers, it was not considered feasible to include search of more Urdu newspapers. Pilot search also helped to ensure that data was being extracted using same categories by the different team members and any confusions were clarified.

For all newspapers, we looked at all e-papers (with every page of all cities online editions) and details of online version of all suicide reports for data extraction. For this study, we operationalized a suicide case as completed suicide by any individual. Cases were excluded in the study if (a) the incidents were not from Pakistan, (b) it was an attempted suicide, (c) suicides in homicide-suicide cases, (d) suicide cases related to terrorism or bombing (although none was noticed in the study period), and (e) if the reports were unclear as to whether the cases were of suicide or of murder.

After operationalizing the suicide case definition, four of the authors independently reviewed one newspaper each for reports of suicides during the study period and extracted data about various sociodemographic characteristics, methods of suicide, possible motives, any suicide note left etc. Then each person entered the data in a spreadsheet designed specifically for this purpose. One of the senior authors (NI) subsequently rechecked and edited all entries in spreadsheet to avoid double reporting of suicide cases. Two of the senior authors (SN & IIH) again double checked the data for any clarifications needed/ mistakes.

Descriptive statistics were used to analyze the data extracted from the newspaper reports, using SPSS 26. Methods of suicide among gender was compared using chi square test and P value <.05 was considered as statistically significant. Current manuscripts describe results from the subset of adolescents’ suicides only.

## RESULTS

Our search yielded 2295 suicide death reports published from January 1, 2020, to December 31, 2021, from the online archives of all selected newspapers. Among these, 289 (13%) were of adolescents (10-18 years old). One hundred fifty one reports were in 2019 and 139 in 2020.

### Demographic Information:

**Fig.1:**
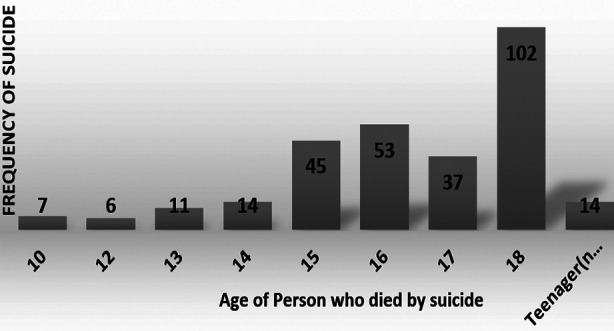
Frequency of Suicide at different ages in newspaper reports.

**Fig.2:**
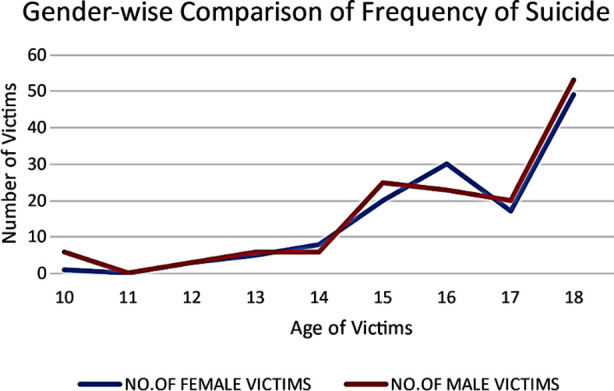
Gender wise comparison of suicide frequency at different ages.

Of the 289 suicides of children and adolescents identified, 149 (51.5%) were males. Age group 15-18 years comprised two thirds (192, 66%) of all suicides (Table-I, Fig.1). There were no suicides in ages<10 years. Fig.2 shows gender wise comparison of suicide at different ages. Highest frequency of suicides was reported from Punjab province (77%) (Table-II).

**Table-I:** Suicide Victims Demographic and relevant details. (N=289).

Variable	Number	%
***Age Categories (n=275)***		
<=15 years	83	30.18
15-18 years	192	69.82
Teenager written in 14 reports, exact age not given.		
***Gender***		
Male	149	51.5
Female	140	48.5
***Education(n=27)***		
School student	21	77.78
College student	5	18.5
Seminary student	1	3.7
***Occupation(n=40)***		
Student	27	67.5
Semi- skilled workers	10	37.03
Unemployed	3	7.5
History of mental illness mentioned	4 (2 among them had Intellectual disability)	1.38

% Are based on available data only.

**Table-II:** Suicide Incident Characteristics (N= 289).

	N	%
***Province***		
Punjab	224	77.5
Sindh	45	15.5
KPK	11	3.80
Baluchistan.	9	3.11
***Area (n= 226)***		
Urban	125	55.3
Rural	101	44.7
***Location(n=81)***		
Inside home	58	71.6
Outside home	23	28.4
***Method of suicide (n=279)***		
Ingestion of Poisonous substance	140	50.17
Unspecified poisonous substance	84	30.10
Kala Pathar	19	6.81
Wheat pills	13	4.65
Pills (Unspecified)	11	3.94
Bleach/ Acid/ chemical	11	3.94
Rat killing pills	2	0.71
Hanging	97 (8 by hanging with a tree)	34.8
Firearm/shooting	22	7.9
Drowning	16 (4 by jumping in water well)	5.73
Self-immolation	3	1.07
Jumping in front of moving train	1	0.3
***Motive for Suicide (n= 256)***		
Domestic Conflicts	165	64.45
Relationship issues	24	9.4
Financial Reasons/Poverty.	12	4.7
Mental illness	4	1.56
Bereavement	4	1.56
Exam failure/ Academic stress/ less marks	4	1.56
Rape	2	0.8
Being blackmailed after sexual assault	3	1.2
Physical ill Health	1	0.4
Scolding by employer	1	0.4

### Place of suicide:

Among the 81 suicides, for which information was available, majority of decedents were discovered dead within their home, accounting for 58 (71.6%) cases (Table-II).

### Methods of suicide:

The preferred method of suicide in our cohort was ingestion of poisonous substances used by 140/279 (50%) of decedents. Details of ingested substance are mentioned in Table-II. Hanging was the second most common method of suicide in children and adolescents in our study (97, 35%), followed by firearms (22, 7.5%) (Table-II). Out of ninety-seven cases of hanging, eight cases were by hanging from a tree near the house, while all others were completed in rooms at home. Most of decedents used ceiling fans as the suspension point. The other few points of suspension mentioned were gas pipe in kitchen and curtain pole.

The suicide methods used according to Gender is shown in Table-III. Although ingestion of poisonous substances was the most common method in both boys and girls, 60% (88) boys used violent methods (hanging, shooting, drowning, burns and jumping in front of train) compared to 36.4% (51) girls. Compared to the methods of suicide used by males, it is notable that poisoning was significantly over-represented in females children and adolescents, whereas suicide by hanging and firearms were significantly less frequent and under-represented in females (P< 0.001) (Table-III).

**Table-III:** Comparison of Gender and Method of Suicide.

Method used for suicide	Gender		

	Male	Female	Total

	n (%)	n (%)	n (%)
Poisoning *	56 (37.6)	84 (60)	140 (48.4)
Hanging *	60 (40.3)	37 (26.4)	97 (33.6)
Firearms *	17 (11.4)	5 (3.6)	22(7.6)
Drowning	7(4.7)	9(6.4)	16(5.5)
Self-immolation	3(2.6)	0(0)	3(1)
Jumping in front of moving train	1(0.7)	0(0)	1(0.3)
Missing Information	5(3.4)	5(3.6)	10(3.5)

Total	149(51.6)	140(48.4)	289(100)

* P value statistically significant <0.001.

### Reasons for suicide:

Among the assigned reasons suggested by the newspaper reports for suicidal behavior, the most common reason was “domestic conflicts” reported in 65% of cases (n=165) followed by relationship issues in 9.3%, (n=24). Among the domestic conflicts, parental scolding was specifically mentioned in forty-three reports and parental refusal to allow playing PUB-G game in one news report. Relationship issues included Parental refusal to marry person of choice (15), failure in Love affair (7), breakup of engagement (1) and being under pressure to end love marriage (1). Other reasons for suicide in children and adolescents are mentioned in Table-II.

### Psychiatric history:

In four newspaper reports, history of psychiatric illness was mentioned in suicide victims with two having Intellectual disability and depression in two reports. None of the reports mentioned any history of alcohol/ substance abuse, neither any past self-harm attempts.

### Suicide Note:

‘Suicide note’ referred to any form or act of expressing suicidal ideation or intent, including communication through mobile phone applications and internet-based mediums such as Facebook or WhatsApp. Five decedents (four males and one female) left suicide notes either through written message (3) or through mobile phone (2). In written notes one stated “sorry for……. deceased name”, another giving “circumstances of being blackmailed and that she did not want to die” and third one stating that “PUB-G is a killer game”, one decedent called a girl on video call and apologized to his family and his PUB-G partner mentioning that it is a killer game”. Another boy took his own life on camera during video call to girlfriend after sending her picture of a piece of cloth and expressing intention to self-harm.

## DISCUSSION

Studies addressing suicide in Pakistan are sparse and to the best of our knowledge no previous studies have investigated suicide in children and adolescents in the country. Our study included suicide deaths in children and adolescents ages <18 which is significant as suicide data in WHO do not discriminate suicide rates and trends in children and adolescents.

Suicide is a rare event in young children however increases in frequency in later adolescence. Two-thirds of age group in our study was 15-18 years. These results are in keeping with previous studies reporting sharp rise in suicide rates in adolescence around 15 years.^13^ There is little empirical data to explain the direct relationship between age and incidence of suicide in youth. The younger children are shielded by parents from exposure to many stressors faced by older adolescents and are likely to have less cognitive maturity and practical ability to plan and carry out suicide.^14^ Increase in incidence of suicide in late adolescence substantiates the need for suicide prevention interventions in this age group.

Suicide in adolescents also varies significantly between genders with higher suicide rates reported in males.^2^ In present study, we noted minimal difference in suicide reports according to gender with 48.4% females to 51.5% male suicides like previous literature from Asia.^15^ This gender difference is explained by different societal and cultural expectations in Asian societies compared from the West. Women may face more societal pressures but men aren’t allowed any latitude for emotional expression thus making them unlikely to seek help during stress.^16^

There are gender and age-specific differences in the methods of suicide used.^16^ Availability and socio-cultural acceptability of lethal methods also has a significant impact on the method of suicide used.^13^ Ingestion of poisonous substances was the method of choice for suicide in our cohort. This finding contrasts with previous research in which the most prevalent methods of suicide in 0-19 years were hanging, jumping from heights and railway-suicides (both genders), intoxication (females) and firearms (males).^13^,^17^ This is not surprising as intake of substances like pesticides, wheat pills & paraphenylenediamine (Kala Pathar) may be aggravated by low cost, ease of access, and lack of regulations on purchase of pesticides etc. by young people without justifying needs to adults. Similarly bleach and corrosives are readily available in homes. Ceiling fans in homes are easily accessible due to hot weather and can translate to impulsive suicide. The relatively high proportion of firearm suicides in our cohort is also alarming. It is unlikely that children and adolescents who completed suicide by firearms own firearms themselves. Thus, the availability of firearms in households seems to be a risk factor not only for the gun owner, but also for other family members. Drowning as a means of suicide, appears to be overrepresented in our cohort, contrary to previous literature possibly due to ease of access with canals and rivers being easily accessible, and swimming lessons being rare.^18^

Suicide in children and adolescents is the result of a complex interplay between genetic, biological, psychological, and social factors.^19^ Disharmony in family environment such as parent child conflicts, low perceived support and lack of family cohesion are risk factors for adolescent suicidality.^17^ In our cases, suicide act tended to be acutely precipitated by arguments or scolding by parents in at least forty-three victims. After domestic conflicts, relationship issues was noted as second most common precipitant for suicide in our sample. Children in Pakistani society are mostly encouraged to submit to parental authority in all spheres of life. It is also possible that parents felt that their children were too young to assume responsibilities of a marriage or considered a pre-marital relationship a moral disrepute.

Only four reports had presence of developmental and psychiatric disorders in our study that is in stark contrast with psychological autopsy studies reporting psychiatric disorders in 81-95% of child and adolescent suicides.^14^,^20^ This is not surprising as psychiatric disorders in children and adolescents often remain undetected and untreated in LMICs.^21^

A recent systematic review has suggested that gaming platforms like PUBG highly influences the presence of suicidality, self-harm, and aggressive behaviours.^22^ Therefore, there is urgent need for research in understanding the vulnerability factors of young adolescents (including mental health problems) and their contribution to dysfunctional effects of online games including suicide.

Although preliminary in nature, the results of the present study have a number of implications for clinicians and researchers working with children and adolescents First and foremost, there is utmost urgency to restrict access to highly lethal means.^13^ Enhancing parenting skills, helping them to improve their communication patterns with their children and adolescents , heath education programs to address stigma around mental illness and suicide,, can be helpful.^23^ Future initiatives should also include mental health literacy and life skills trainings for adolescents in schools, teacher trainings, to identify at risk students, psychosocial interventions for at risk population, social media use in prevention campaign and setting up of helplines and access to professional expertise for early intervention and treatment.^18^,^23^

### Limitations of the study:

The present study findings should be interpreted in the light of several limitations. First the number of suicide reports in newspapers in present study is a gross underestimation of the child and adolescent suicide in Pakistan due to steps taken by families to prevent mention of suicide as a cause of death, stigma, suicide and attempted suicide still being a criminal act in Pakistan punishable under law etc. Our findings are also limited by the fact that most of crime reporters/ journalists base their reports on verbal account of police at the scene of crime and lack information about mental health history as well as an account of risk factors and protective factors.. Another limitation is that study was partially conducted during COVID Pandemic, which had a huge impact on mental health of masses including adolescents. Information about victims suffering from COVID-19 or having experienced loss of loved ones could have been an important variable but was not studied due to missing information.^24^

Despite these limitations, our study provides useful insights into children and adolescent suicides to establish trends, to understand some risk factors and other correlates for suicide prevention and risk reduction and intervention strategies in Pakistan.

## CONCLUSION

A large number of suicides are reported in children and adolescents in Pakistan with high incidence in late adolescence. The predominant method of suicide in this group was ingestion of poisonous substances. Domestic conflicts including arguments with parents were identified as commonest acute precipitant in our cohort. Suicide prevention strategies should be tailored to address local trends and observations as well as actively involve all stakeholders including families and school authorities to help vulnerable children and adolescents. This study serves as baseline for future approaches to suicide research aimed at deeper examination of the suicide phenomenon in children and adolescents in Pakistan to inform prevention programmers. There is an urgent need for broader sociodemographic and epidemiological enquiry into the adolescent suicide phenomenon aimed at uncovering the subtleties regarding causes, risks, protective factors, and prevention, in the future.

## Availability of data and materials:

All data generated or analyzed during this study are included in this published article. All selected newspapers editions, on which the findings of the paper rely, are publicly available to readers on Newspapers websites.
